# Circadian rhythm-based prognostic features predict immune infiltration and tumor microenvironment in molecular subtypes of hepatocellular carcinoma

**DOI:** 10.1515/biol-2025-1208

**Published:** 2026-01-01

**Authors:** Jinhai Wang, Li Ma, Jinjuan Wang, Zhe Ma, Weiwei Wang, Songning Yu

**Affiliations:** Department of Hepatobiliary Surgery, General Hospital of Ningxia Medical University, Yinchuan City, China; Department of Respiratory and Critical Care Medicine, The Fifth People’s Hospital of Qinghai Province, Xining, China; Ningxia Medical University, Yinchuan, China

**Keywords:** circadian rhythm, prognostic features, hepatocellular carcinoma molecular subtypes, immune infiltration, tumor microenvironment, lncRNA AC019080.1

## Abstract

Hepatocellular carcinoma (HCC) poses a significant threat to human health. Tumor microenvironment alterations, particularly immune-related changes, play a pivotal role in HCC progression, with high-throughput technologies facilitating the exploration of these dynamics. This study aimed to investigate the role of long non-coding RNA (lncRNA) AC019080.1 in HCC cells. A total of 24 circadian rhythm-related (CRR) messenger RNAs (mRNAs) and 433 CRR-lncRNAs were identified. Among them, 46 prognostically relevant circadian rhythm-related lncRNAs (PCRR-lncRNAs) were found to be upregulated in the HCC group. Molecular clustering analysis of 370 HCC samples revealed expression differences of PCRR-lncRNAs across three subtypes. Immune cell infiltration levels and tumor microenvironment analysis revealed significant subtype-specific differences. AC019080.1 and MCM3AP-AS1 were identified as core PCRR-lncRNAs in HCC, with elevated expression in both HCC tissues and cell lines. Through suppression of the Wnt/β-catenin signaling pathway, knockdown of lncRNA AC019080.1 significantly inhibited the proliferation, colony formation, migration, and invasion of HCC cells, while promoting apoptosis. This study suggests that circadian rhythm-related genes can predict immune infiltration and the molecular subtypes of HCC, providing valuable insights for diagnosis and treatment. lncRNA AC019080.1 holds potential as a therapeutic target for HCC.

## 1 Introduction

Hepatocellular carcinoma (HCC) is the most common form of primary liver cancer, accounting for approximately 90 % of all liver cancers worldwide, with high incidence and mortality rates [1]. Risk factors such as hepatitis B virus (HBV), hepatitis C virus (HCV), alcoholic liver diseases, and the increasingly prevalent non-alcoholic steatohepatitis (NASH) often contribute to late-stage diagnosis, as early HCC typically presents few clinical signs and symptoms [2]. Many patients are diagnosed at an advanced stage, missing the optimal surgical window, resulting in limited treatment options and a high risk of metastatic recurrence despite conservative therapies [3]. The 5-year survival rate for early-stage HCC is around 70 %, while the median survival for advanced-stage patients is merely 1–1.5 years [4], underscoring the urgent need for early diagnosis to improve treatment outcomes and patient survival. Existing diagnostic methods have significant limitations. Alpha-fetoprotein (AFP), the primary serum marker for screening, has low sensitivity and unreliable results [5]. Histopathological analysis, considered the “gold standard” for tumor diagnosis, has limited applicability in liver cancer detection due to high costs and limited access to tumor tissues [6].

High-throughput sequencing technology and bioinformatics have opened new avenues for understanding tumors. With decreasing sequencing costs and increasing sequencing depth, large-scale high-quality data can now be obtained, enabling the exploration of potential diagnostic, therapeutic, and prognostic targets [7]. Databases like The Cancer Genome Atlas (TCGA), co-founded by the National Human Genome Research Institute (NHGRI) and the National Cancer Institute (NCI) in 2006, which includes molecular characterization of over 20,000 primary malignant tumors and normal samples from 33 cancers along with comprehensive clinical data, play a vital role in data storage and analysis [8], [9], [10], [11].

The circadian rhythm is a cellular system that aligns the body’s metabolism with environmental light-dark cycles [12]. Regulated by central and peripheral molecular clocks, it operates through a transcriptional-translational feedback loop (TTFL) at the molecular level [13], [14], [15], [16]. As a central metabolic organ, the liver exhibits circadian rhythms in over 50 % of its metabolites [17]. Disruptions to circadian genes, whether genetic or environmental, can lead to hepatic metabolic disorders and worsen liver pathology, influencing processes such as glucose, bile acid, and fatty acid metabolism [18]. Non-coding RNAs, including microRNAs (miRNAs) and long non-coding RNAs (lncRNAs), play pivotal roles in regulating circadian rhythms and are implicated in the pathogenesis of HCC [19], 20]. MiRNAs regulate genes post-transcriptionally, and studies in mutant mice suggest that circadian-regulated miRNAs may contribute to liver cancer by modulating genes involved in cell proliferation, invasion, and metabolism [21]. LncRNAs, such as LncRNA-HULC, are upregulated in HCC tissues and positively correlated with the Clock activator, promoting HCC cell growth both *in vitro* and *in vivo* [22]. Risk factors for HCC, including chronic viral hepatitis, long-term carcinogen exposure, and diabetes, are associated with circadian rhythm disruptions [23]. Aberrant expression of circadian genes in HCC, such as the suppression of Per and Cry, disrupts the cancer cell circadian rhythm, promoting cancer cell survival and carcinogenesis [24], 25].

This study analyzed transcriptomic data from TCGA and identified associations between HCC, 24 circadian rhythm-related (CRR) mRNAs, and 433 CRR-lncRNAs. Immune cell infiltration and tumor microenvironment (TME) analyses, alongside quantitative real-time polymerase chain reaction (qRT-PCR), revealed significant positive co-expression of AC019080.1 with other prognostic CRR-lncRNAs. Our study aims to comprehensively explore the role and mechanism of lncRNA AC019080.1 in HCC cells by comparing its expression in normal and cancer cells and conducting loss-of-function assays. These findings could enhance our understanding of HCC molecular mechanisms and offer a novel lncRNA-based target for diagnosis and treatment.

## 2 Methods

### 2.1 Data collection and organization

Transcriptome expression data and clinical information for HCC samples were obtained from the TCGA database (https://tcga-data.nci.nih.gov/tcga/), a comprehensive cancer gene expression resource that includes 374 HCC tissue samples and 50 normal control (NC) tissue samples. Custom Perl and R scripts were utilized to clean and organize the data for subsequent bioinformatics and statistical analysis.

### 2.2 Identification of circadian rhythm-related lncRNAs by co-expression analysis

First, RNA categorization was performed on the transcriptome expression matrices of all HCC and NC samples to differentiate between mRNAs and lncRNAs. Subsequently, 24 CCR genes (CRRGs) were identified from the literature (PMID: 37183243 and PMID: 37373286), and the R package limma was used to extract the relative expression of these CRR-mRNAs from the gene expression matrix. Co-expression analysis of CRR-mRNAs with lncRNAs was subsequently performed using the Pearson correlation test to identify CRR-lncRNAs. A Pearson correlation coefficient of > 0.5 and a *P*-value of < 0.05 were considered statistically significant.

### 2.3 Identification of prognostically relevant CRR-lncRNAs in HCC

The expression matrix of CRR-lncRNAs was merged with clinical data (survival time and survival status) for patients with HCC. The R package survival was then used to identify prognostically relevant CRR-lncRNAs (PCRR-lncRNAs) through one-way Cox regression analysis. Differential expression analysis of PCRR-lncRNAs between HCC and NC groups was performed using the R package limma, and the results were visualized using box-and-whisker plots and heatmaps. A *P*-value < 0.001 was considered statistically significant.

### 2.4 Molecular cluster analysis of HCC samples

Based on the expression values of PCRR-lncRNAs, the R package ConsensusClusterPlus was employed to perform molecular clustering analysis on all patients with HCC. The maximum number of clusters (k) was set to 9, the clustering algorithm was set to “km,” and the similarity of the samples was assessed using the “Euclidean” distance. The optimal number of clusters (k) was determined by analyzing the cumulative distribution function (CDF) curve, cluster consensus scores, and the consensus matrix. Survival differences between patients with different molecular subtypes were analyzed using the survival and survminer R packages, and visualized using survival curves. Finally, the expression differences of PCRR-lncRNAs between patients with different molecular subtypes were explored, and clinical trait differences across subtypes were examined, with the results visualized in a heatmap.

### 2.5 Immune cell infiltration and microenvironmental analysis between different HCC molecular phenotypes

Based on the R package limma, immune cell infiltration analysis was performed on all HCC samples using algorithms including TIMER, CIBERSORT, CIBERSORT-ABS, QUANTISEQ, MCPCOUNTER, XCELL, and EPIC. The infiltration levels of immune cells in samples with different HCC molecular subtypes were analyzed. Subsequently, using the R package estimate, TME analysis was conducted on all HCC samples, calculating the StromalScore, ImmuneScore, and ESTIMATEScore for each sample. Differences in these scores across different molecular subtypes were compared, and the results were visualized using heatmaps and box-and-whisker plots. A *P*-value < 0.05 was considered statistically significant.

### 2.6 Differential expression analysis and correlation analysis of core PCRR-lncRNAs

The top two genes with the highest hazard ratio (HR) from the PCRR-lncRNAs were selected as core PCRR-lncRNAs. Differential expression analysis of these core PCRR-lncRNAs was performed between the HCC group and the NC group, as well as among different molecular subtypes, using the R package limma, and the results were visualized through box-and-whisker plots. Co-expression correlation analysis between the core PCRR-lncRNAs and other PCRR-lncRNAs was performed using the corrplot package in R, and the correlation matrix was visualized.

### 2.7 Clinical sample collection

A total of 25 paired HCC and adjacent non-tumor tissues (collected within 2 cm of the tumor margin) were obtained from patients who underwent surgical resection for HCC between May 2024 and May 2025 at the General Hospital of Ningxia Medical University. Patients were included if they had histologically confirmed primary HCC, no prior anti-tumor treatment (such as chemotherapy, radiotherapy, or targeted therapy), and complete clinical data with written informed consent. Exclusion criteria included the presence of other primary malignancies, severe comorbidities (e.g., decompensated cirrhosis or major organ dysfunction), or incomplete clinical records. Fresh tissue samples were snap-frozen in liquid nitrogen within 30 min after resection and stored at −80 °C until RNA extraction.

**Informed consent:** Informed consent has been obtained from all individuals included in this study.

**Ethical approval:** The research related to human use has been complied with all the relevant national regulations, institutional policies and in accordance with the tenets of the Helsinki Declaration, and has been approved by the Ethics Committee of the General Hospital of Ningxia Medical University.

### 2.8 Cell culture and treatment

The cell lines L-02 (normal) and Huh7, Hep3B, and HepG2 (HCC) were selected for this study. These cell lines were obtained from specialized cell banks, and their identities were authenticated using short tandem repeat (STR) profiling. After acquisition, the cells were cultured in high-glucose Dulbecco’s Modified Eagle Medium (DMEM) (HyClone, No. SH30243.01) containing 10 % fetal bovine serum (FBS, Gibco, No. 10099-141) and 1 % double antibody (penicillin-streptomycin mixture, HyClone, No. SV30010) in a humidified incubator at 37 °C with 5 % CO_2_ (Thermo Fisher Scientific). To induce the Wnt/β-catenin pathway, Huh7 and Hep3B cells were treated with 40 μM SKL2001 (MedChemExpress LLC, Monmouth Junction, NJ, USA) for 1 h.

### 2.9 Cell transfection

Gemma Genetics, a professional company, was commissioned to construct small interfering RNA (siRNA) sequences targeting lncRNA AC019080.1 (si-#1 and si-#2), with a non-targeting control siRNA (si-NC) serving as the negative control. SiRNAs (si-#1, si-#2, or si-NC) were transfected into Huh7 and Hep3B cells during their logarithmic growth phase using Lipofectamine 3,000 transfection reagent (Invitrogen, No. L3000015). One day prior to transfection, the cells were seeded into 6-well plates at an appropriate density to achieve 30 %–50 % confluence at the time of transfection. The transfection protocol was as follows: 5 μL of Lipofectamine 3,000 was mixed with 250 μL of Opti-MEM medium (Invitrogen, item no. 31985062) and incubated for 5 min at 25 °C. Meanwhile, 10 pmol of siRNAs (si-#1, si-#2, or si-NC) was mixed with 250 μL of Opti-MEM medium. The two mixtures were gently combined and incubated at 25 °C for 20 min, after which they were added dropwise to the wells of the 6-well plates and incubated for an additional 6–8 h. The cells were then used for subsequent experiments.

### 2.10 Cell counting kit-8 (CCK-8) assay

Huh7 and Hep3B cells were seeded into 96-well plates at a density of 5 × 10^3^ cells per well, with five replicate wells per group. At 0, 24, 48, and 72 h post-transfection, 10 μL of CCK-8 reagent (Dojindo, CK04) was added to each well, and the cells were incubated for 1–4 h at 37 °C. The optical density (OD) was measured at 450 nm using a Bio-Tek Epoch2 microplate reader (Bio-Rad Laboratories, USA). Cell viability curves were generated based on the OD values to assess cell proliferation over time.

### 2.11 Colony formation assay

The clonogenic potential of Huh7 and Hep3B cells was evaluated by the colony formation assay. Cells were seeded at low density (500 cells/well in 6-well plates) and cultured in complete DMEM (10 % FBS) for 14 days, with medium changes every 3 days. Colonies were fixed with 4 % paraformaldehyde, stained with 0.1 % crystal violet, and those containing > 50 cells were counted under a light microscope (Axio Observer A1; Carl Zeiss, 40 × magnification). Three independent experiments were performed.

### 2.12 Transwell assay

In the migration assay, 200 μL of transfected cells were added to the upper chamber of a Transwell (Corning, No. 3422, 8.0 μm pore size polycarbonate membrane), with the lower chamber filled with 600 μL of medium containing 20 % FBS. After incubation, non-migrated cells in the upper chamber were removed, while cells that migrated to the lower surface were fixed with 4 % paraformaldehyde for 15 min, stained with crystal violet for 15 min, washed with phosphate-buffered saline (PBS), and counted under a microscope (Axio Observer A1, Carl Zeiss Shanghai Co., Ltd., Shanghai, China) in five randomly selected fields of view. In the invasion assay, Matrigel matrix gel (BD, No. 354234, 1:8 dilution) was applied to the upper chamber and air-dried at 4 °C overnight. The remaining procedures followed the migration assay, with the incubation time extended to 36 h to assess cell invasion capacity.

### 2.13 Flow cytometry assay

Following transfection, Huh7 and Hep3B cells were collected and resuspended in 500 μL of Binding Buffer (Annexin V-fluorescein isothiocyanate (FITC)/propidium iodide (PI) Apoptosis Detection Kit, BD, No. 556547) at a concentration of 1 × 10^6^ cells/mL. Next, 5 μL of Annexin V-FITC and 5 μL of PI were added to the cell suspension. The cells were incubated for 15 min at 25 °C, protected from light, and analyzed using a BD FACSCalibur flow cytometer within 1 h. The apoptosis rate was calculated using FlowJo software (Version 10; FlowJo, LLC).

### 2.14 Quantitative real-time polymerase chain reaction (qRT-PCR)

Total RNA was extracted using TRIzol reagent (Invitrogen, No. 15596026) from tissues and cells, and RNA concentration and purity were assessed using NanoDrop 2000 (Thermo Fisher Scientific), ensuring an A260/A280 ratio between 1.8 and 2.0. Reverse transcription into cDNA was carried out according to the instructions of the reverse transcription kit (TaKaRa, No. RR047A). Specific primers for lncRNA AC019080.1, MCM3AP-AS1, and GAPDH (used as an internal reference) were synthesized by Sangyo Bioengineering (Shanghai) Co. A 20 μL qRT-PCR reaction system was used on a Bio-Rad CFX96 Touch real-time fluorescence quantitative PCR instrument. The relative gene expression was calculated using the 2^−ΔΔCt^ method based on Ct values.

### 2.15 Western blot

HCC cells (Huh7 and Hep3B) were lysed using RIPA buffer (P0013, Beyotime, Shanghai, China) containing protease and phosphatase inhibitors, and total protein was quantified using a BCA assay (Thermo Fisher Scientific). Equal amounts of protein (20 μg) were separated by SDS-PAGE and transferred onto PVDF membranes. The membranes were blocked with 5 % non-fat milk for 1 h at room temperature, then incubated overnight at 4 °C with primary antibodies against p-PI3K (1:1,000, ab138364), PI3K (1:1,000, ab302958), p-AKT (1:1,000, ab38449), AKT (1:10,000, ab179463), *β*-catenin (1:5,000, ab32572), and GAPDH (used as a loading control) (1:2,500, ab9485). After washing, membranes were incubated with appropriate HRP-conjugated secondary antibodies (1:2,000, ab6721) for 1 h at room temperature. Protein bands were visualized using enhanced chemiluminescence (ECL) reagents (#WBULS0100, Millipore, USA) and detected by a chemiluminescence imaging system. Band intensities were quantified using ImageJ software (NIH, Bethesda, MD, USA), and target protein levels were normalized to GAPDH for semi-quantitative analysis.

### 2.16 Statistical analysis

In this study, various statistical methods and bioinformatics tools were employed to systematically analyze the experimental data. CRR-lncRNAs were identified using Pearson’s correlation test, and PCRR-lncRNAs were selected through one-way COX regression analysis and analyzed for differential expression. Molecular clustering, immune assay, and core PCRR-lncRNAs analysis were conducted with the help of relevant R packages. Cellular experiments, including qRT-PCR and CCK-8, were used to assess relevant parameters, with all analyses performed in R to ensure the accuracy and reliability of the results. All wet-lab experiments were performed in triplicate, and numerical data are presented as mean ± standard deviation (SD). Statistical comparisons between two groups were performed using two-tailed Student’s *t*-tests, while one-way analysis of variance (ANOVA) followed by Tukey’s post hoc test was used for comparisons among multiple groups.

## 3 Results

### 3.1 Identification of CRR-lncRNAs

**Figure 1:**
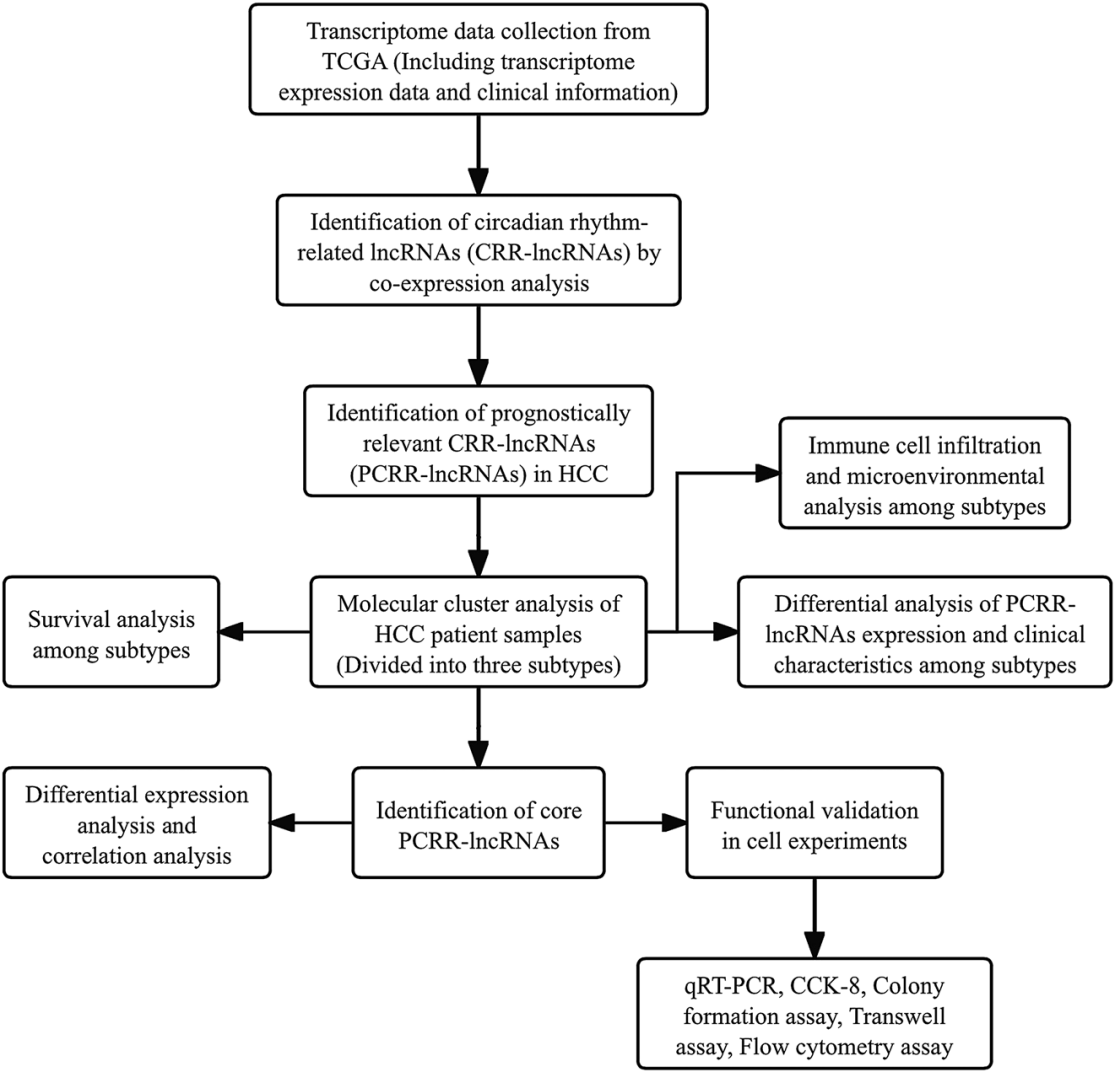
Study flowchart.

**Figure 2:**
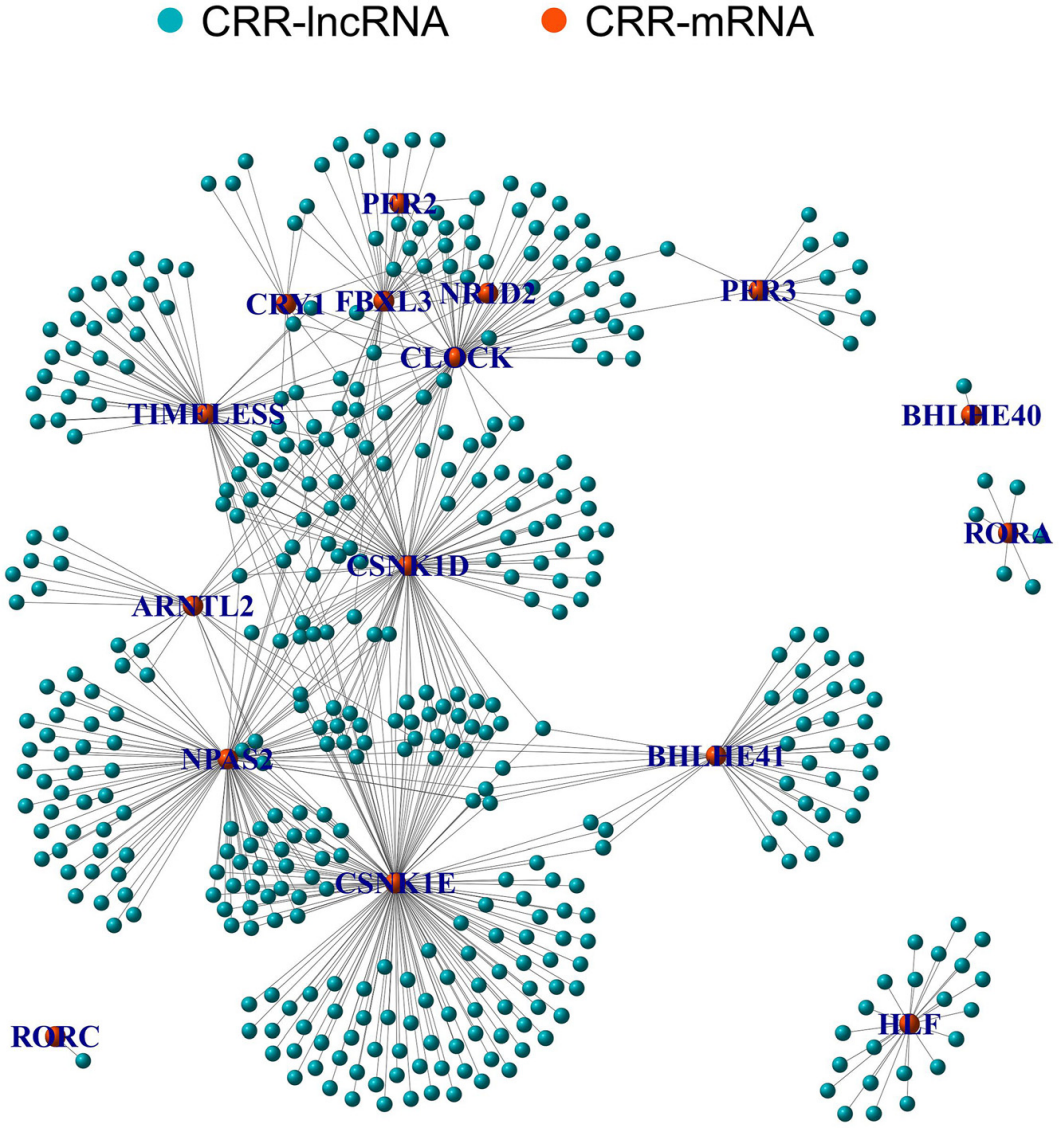
MCo-expression network between CRR-mRNAs and CRR-lncRNAs. Note: CRR, circadian rhythm-related.

The flowchart of this study is shown in Figure 1. A transcriptome expression matrix, including 59,427 RNAs, was collected and organized from the TCGA database (Table S1), comprising 16,773 lncRNAs and 19,895 mRNAs (Tables S2 and S3). A matrix of 24 CRR-mRNAs was constructed based on the mRNA matrix data reported in the literature and in HCC (Table S4). Co-expression network analysis identified 433 CRR-lncRNAs, with their expression matrices and co-expression relationships detailed in Tables S5 and S6. The co-expression networks visualized the correlation between the 24 CRR-mRNAs and the 433 CRR-lncRNAs (Figure 2).

### 3.2 Identification of PCRR-lncRNAs

**Figure 3:**
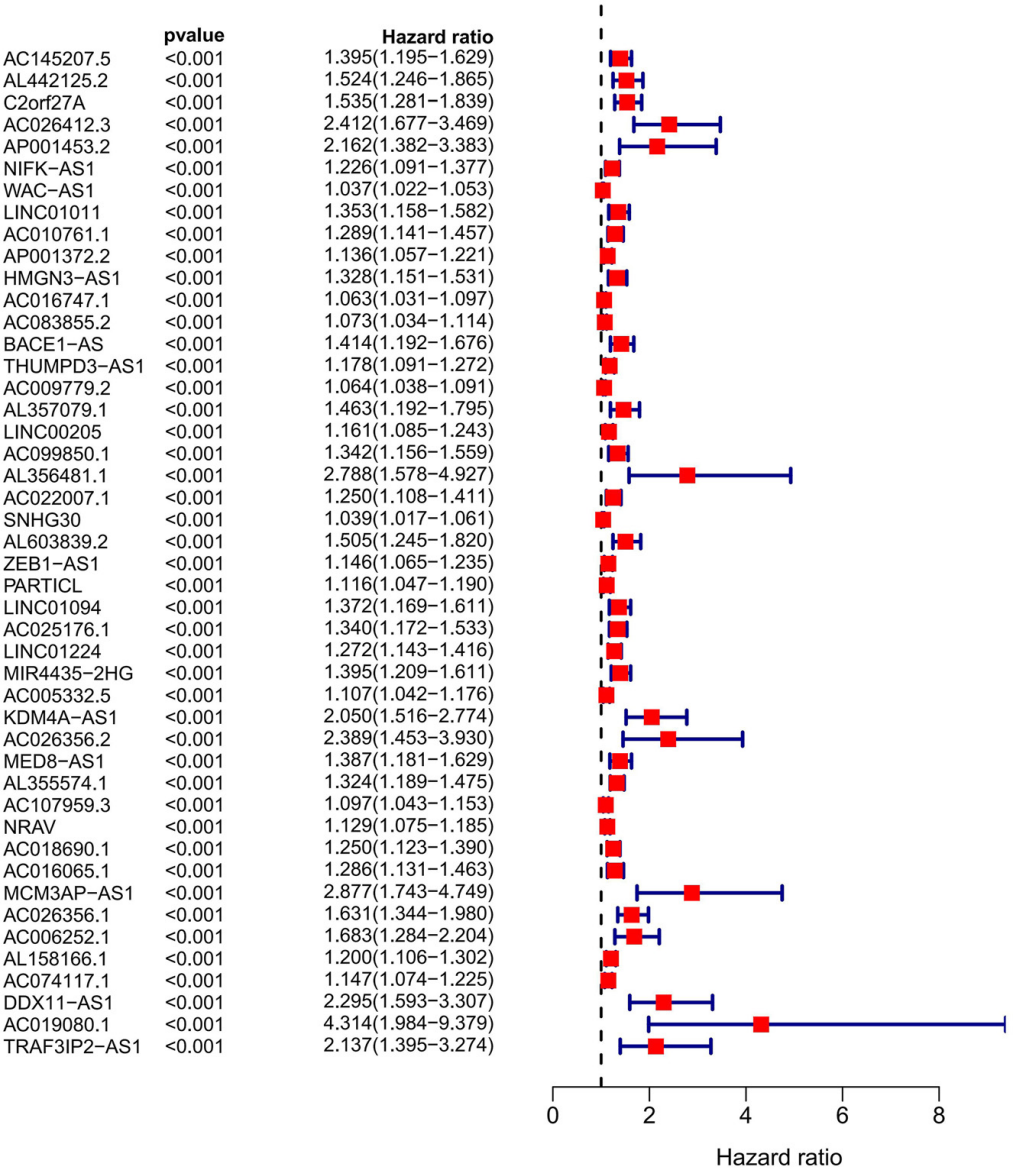
Forest plot showing 46 PCRR-lncRNAs for HCC. Note: HCC, hepatocellular carcinoma.

**Figure 4:**
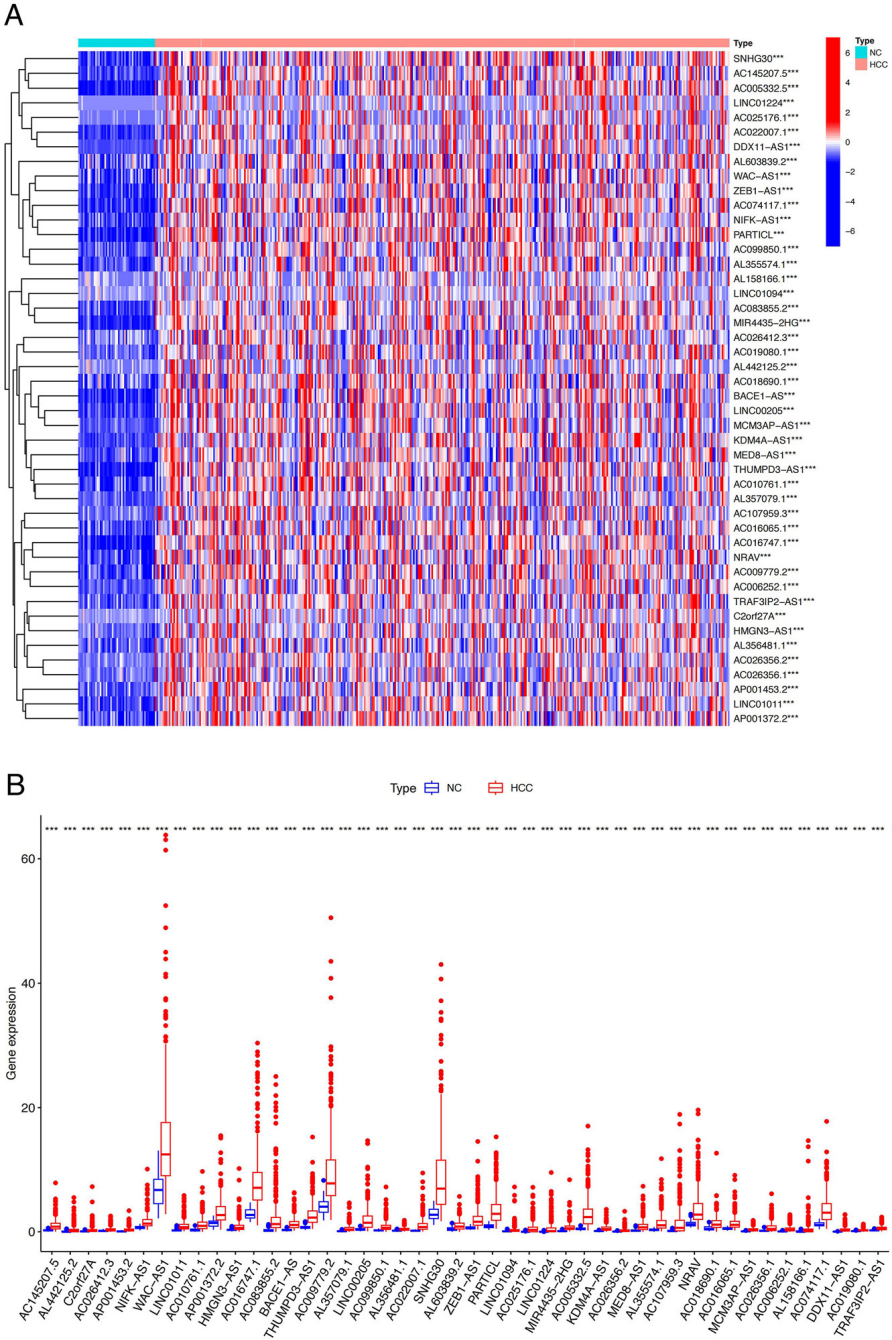
Differential expression of PCRR-lncRNAs. Heatmap (A) and boxplot (B) demonstrating the differential expression of 46 PCRR-lncRNAs between the HCC and NC groups. ^***^ *P* < 0.001. Note: PCRR, prognostically relevant circadian rhythm-related gene; HCC, hepatocellular carcinoma; NC, normal control.

Using one-way COX regression analysis, 46 PCRR-lncRNAs were identified (HR > 1, *P* < 0.001) (Figure 3). All of these PCRR-lncRNAs were upregulated in HCC tissues compared to the NC group (*P* < 0.001) (Figure 4A and B).

### 3.3 Identification of HCC molecular typing

**Figure 5:**
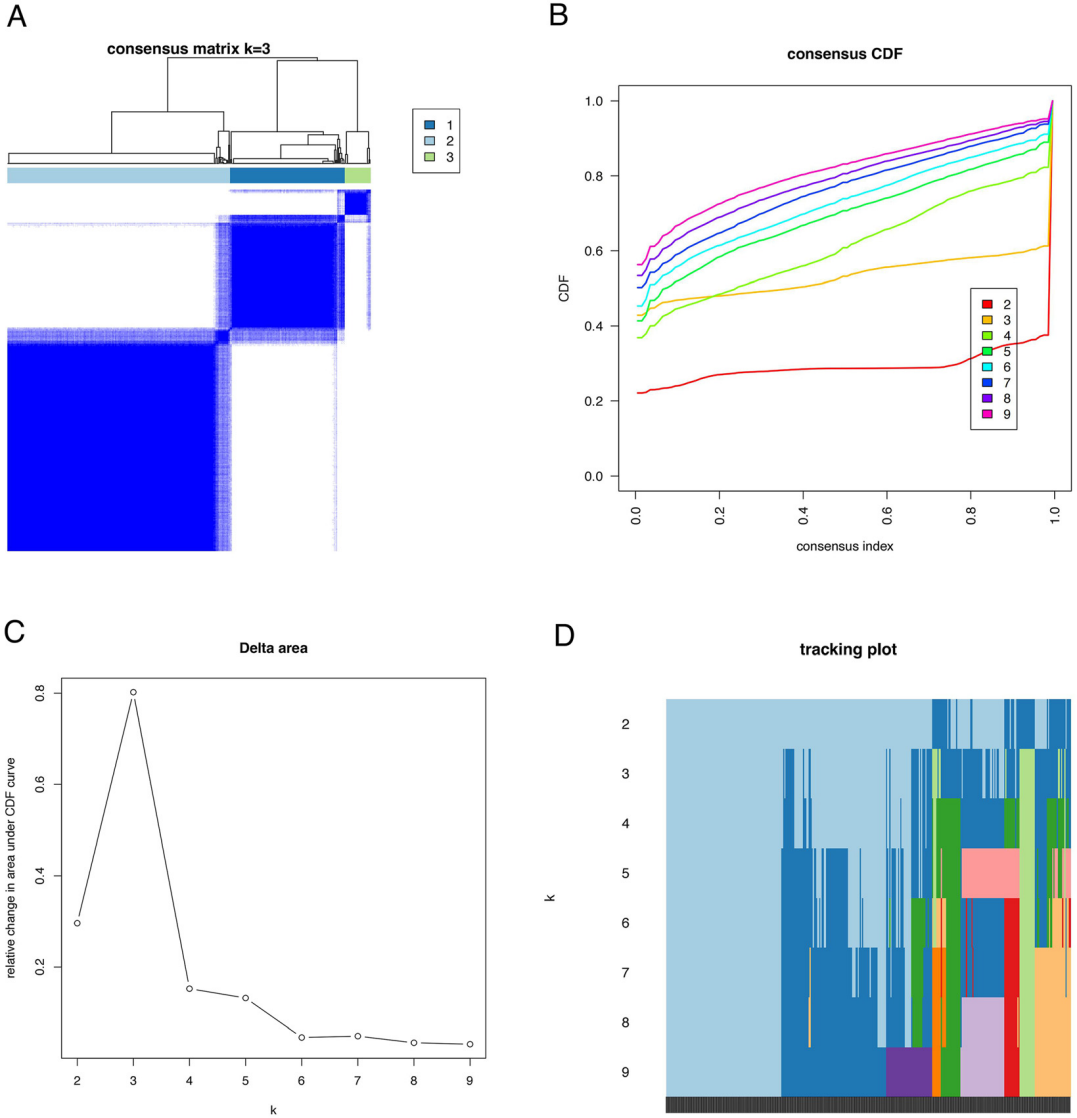
Identification of HCC molecular subtypes. (A) Clustering consensus matrix for *k* = 3. (B) CDF curve at *k* = 2–9. (C) Area under the CDF curve at *k* = 2–9. (D) Tracking plot for *k* = 2–9. Note: CDF, cumulative distribution function.

**Figure 6:**
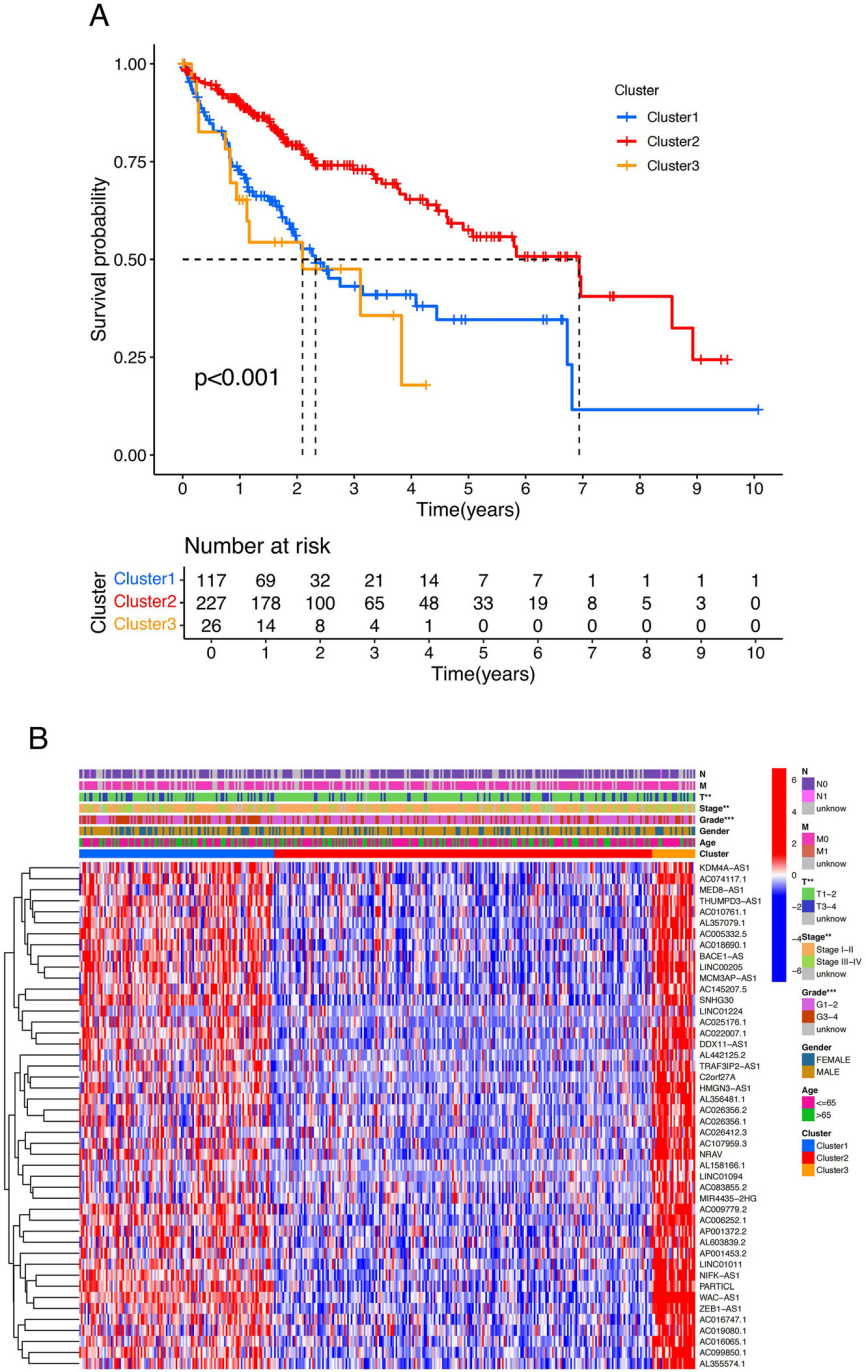
Survival analysis of HCC molecular subtypes. (A) Survival curves demonstrating the difference in survival prognosis among the three groups of HCC subtypes. (B) Heatmap demonstrating the expression differences of PCRR-lncRNAs as well as the distribution of clinicopathologic features among the three groups of HCC subtypes. Note: PCRR, prognostically relevant circadian rhythm-related gene; HCC, hepatocellular carcinoma.

Based on the expression profiles of the 46 PCRR-lncRNAs, molecular clustering analysis was performed on 370 HCC samples (only those with complete clinical data were retained). The cluster stability was highest when k = 3, as indicated by the fluctuation of the CDF curve between 0.2 and 1.0 (Figure 5A and B). The area under the CDF curve for k = 2 to 9 is shown in Figure 5C. Notably, the lowest level of interference between subtypes was observed when k = 3 (Figure 5D). Furthermore, a significant difference in survival prognosis was observed between the three subtypes, with the Cluster2 subtype showing the best prognosis and the Cluster3 subtype the worst prognosis (Figure 6A). The heatmap illustrates differences in the expression of PCRR-lncRNAs and the distribution of clinicopathologic features among the three subtypes (Cluster1, Cluster2, and Cluster3) (Figure 6B).

### 3.4 Differences in immune cell infiltration and tumor microenvironment between the three groups of HCC molecular subtypes

**Figure 7:**
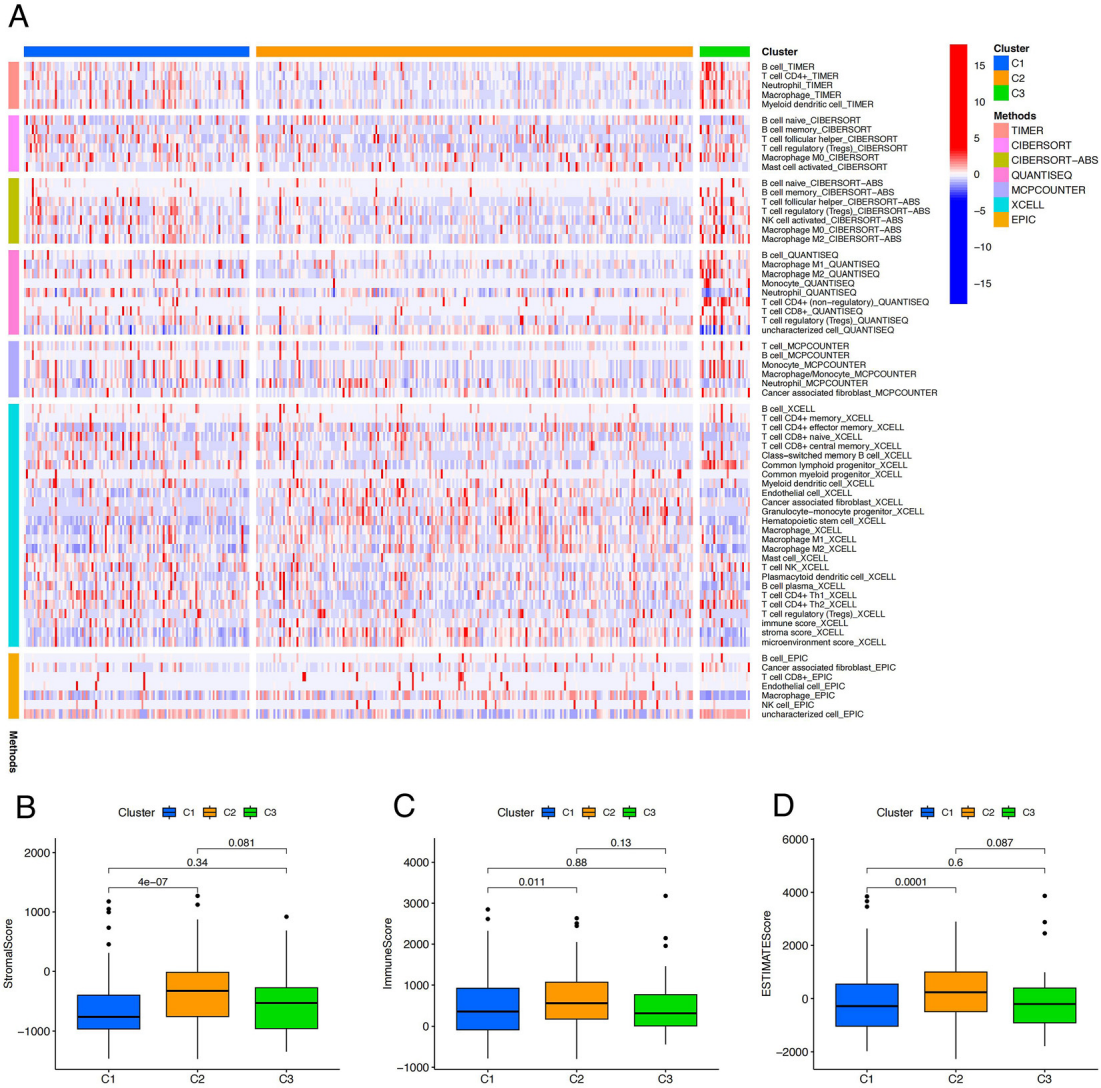
Immune infiltration and microenvironment differences among the three cluster subtypes. (A) Heatmap demonstrating the differences in immune cell infiltration between the three subtypes under different algorithms. (B, C, D) Box line plots demonstrating the differences in StromalScore, ImmuneScore, and ESTIMATEScore between the three subtypes.

Immune cell infiltration levels across the three HCC subtypes were calculated using seven immune cell infiltration analysis algorithms. The heatmap displayed differences in immune cell infiltration between Cluster1, Cluster2, and Cluster3 under the different algorithms. In the majority of algorithms, the C1 subgroup exhibited elevated infiltration levels of B cells (including naive and memory B cells) and macrophages (M0, M1, and M2), indicating a complex immune microenvironment in this subgroup. The C2 subgroup demonstrated higher infiltration of CD8^+^ T cells, especially in the TIMER and EPIC algorithms, suggesting its potential as an optimal candidate for immune checkpoint inhibitor therapy. Meanwhile, the C3 subgroup showed increased infiltration of Tregs, particularly in the TIMER and CIBERSORT algorithms, indicating a robust immunosuppressive environment within this subgroup (Figure 7A). TME analysis revealed that the Cluster2 subtype had significantly higher StromalScore, ImmuneScore, and ESTIMATEScore than the Cluster1 subtype, with statistical differences (*P* < 0.05) (Figure 7A–C).

### 3.5 Identification of the 2 core PCRR-lncRNAs

**Figure 8:**
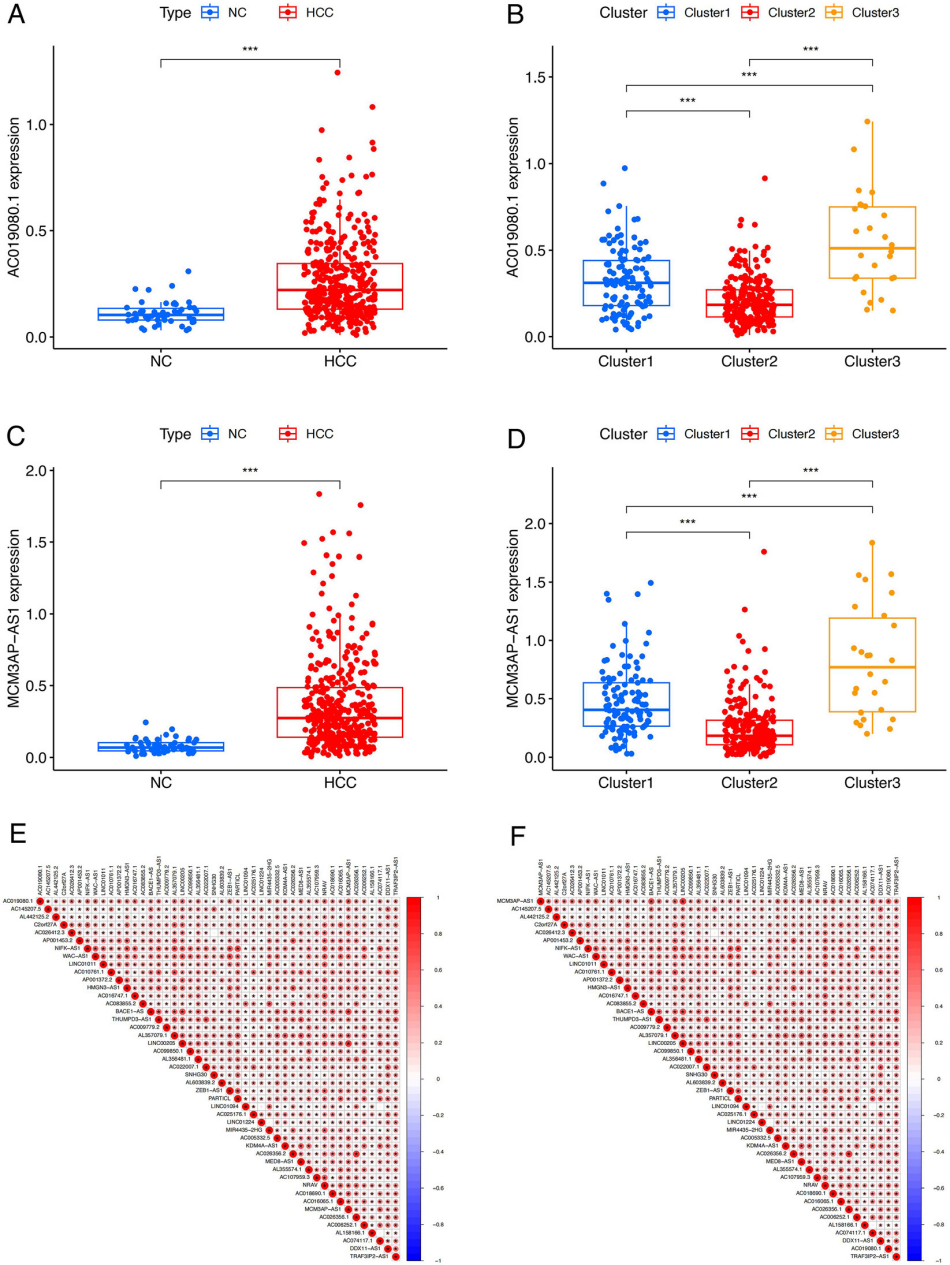
Expression patterns and correlations of core PCRR-lncRNAs. (A) Differential expression of AC019080.1 and (C) MCM3AP-AS1 between HCC and NC groups. (B) Differential expression of AC019080.1 and (D) MCM3AP-AS1 between the three groups of cluster subtypes. (E) Co-expression correlation of AC019080.1 and (F) MCM3AP-AS1 with other PCRR-lncRNAs, respectively. ^***^ *P* < 0.001. Note: PCRR, prognostically relevant circadian rhythm-related gene; HCC, hepatocellular carcinoma; NC, normal control.

Among the 46 PCRR-lncRNAs, the top two lncRNAs with the largest HRs were AC019080.1 (HR = 4.31) and MCM3AP-AS1 (HR = 2.88), representing the core PCRR-lncRNAs in HCC. Both AC019080.1 and MCM3AP-AS1 were significantly upregulated in the HCC group compared to the NC group (*P* < 0.001) (Figure 8A and C). Additionally, the relative expression of AC019080.1 and MCM3AP-AS1 was lowest in the Cluster2 subgroup and highest in the Cluster3 subgroup (*P* < 0.001) (Figure 8B and D). The correlation matrix indicated significant positive co-expression between AC019080.1, MCM3AP-AS1, and other PCRR-lncRNAs (Figure 8E and F).

### 3.6 The level of AC019080.1 and MCM3AP-AS1 Are upregulated in HCC tissues

**Figure 9:**
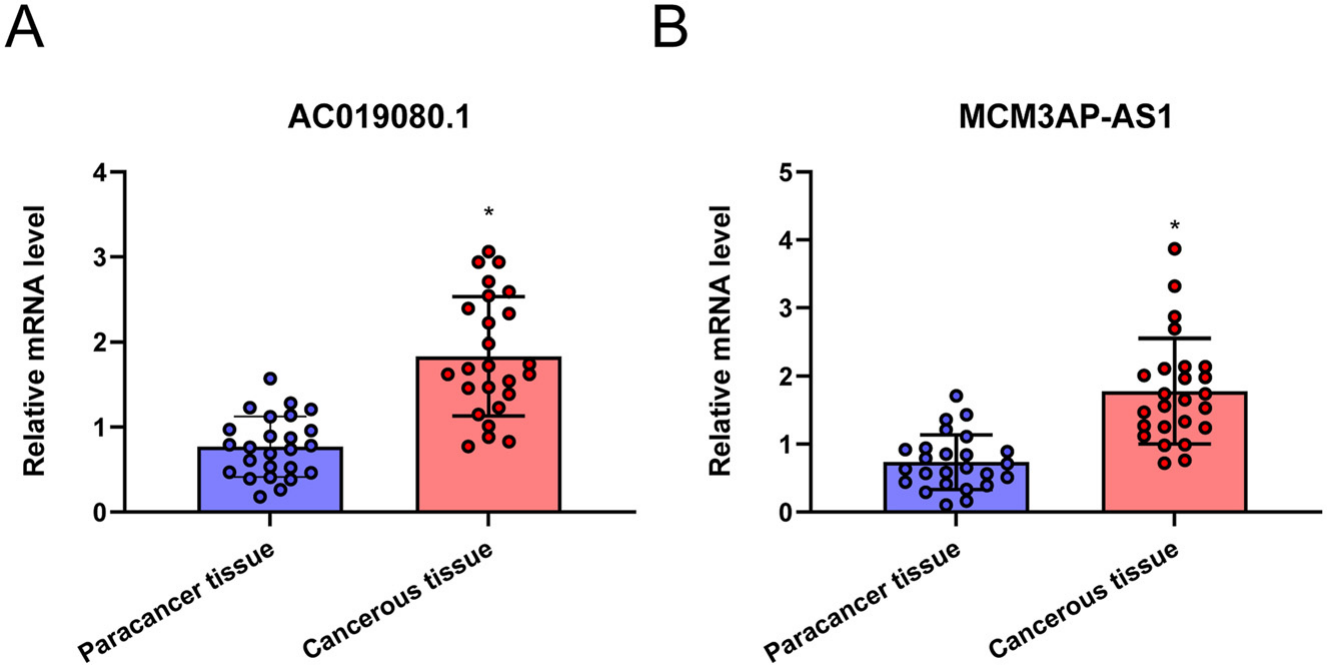
The level of AC019080.1 and MCM3AP-AS1 are upregulated in HCC tissues. qRT-PCR was used to detect the expression levels of AC019080.1 (A) and MCM3AP-AS1 (B) in HCC tissues and paracancerous tissues. Data are presented as mean ± standard deviation (SD) from three independent experiments. ^*^ *P* < 0.05 versus Paracancer tissues. Note: qRT-PCR, quantitative real-time polymerase chain reaction.

To further validate the expression levels of AC019080.1 and MCM3AP-AS1, RNA was extracted from 25 paired HCC tissues and adjacent non-cancerous tissues. qRT-PCR analysis confirmed significantly higher expression levels of both AC019080.1 and MCM3AP-AS1 in HCC tissues compared to paracancerous tissues (*P* < 0.05) (Figure 9A and B). However, since MCM3AP-AS1 has already been studied in HCC [26], and AC019080.1 has not been previously investigated, AC019080.1 was selected for further functional validation.

### 3.7 Knockdown of AC019080.1 inhibits HCC cell progression

**Figure 10:**
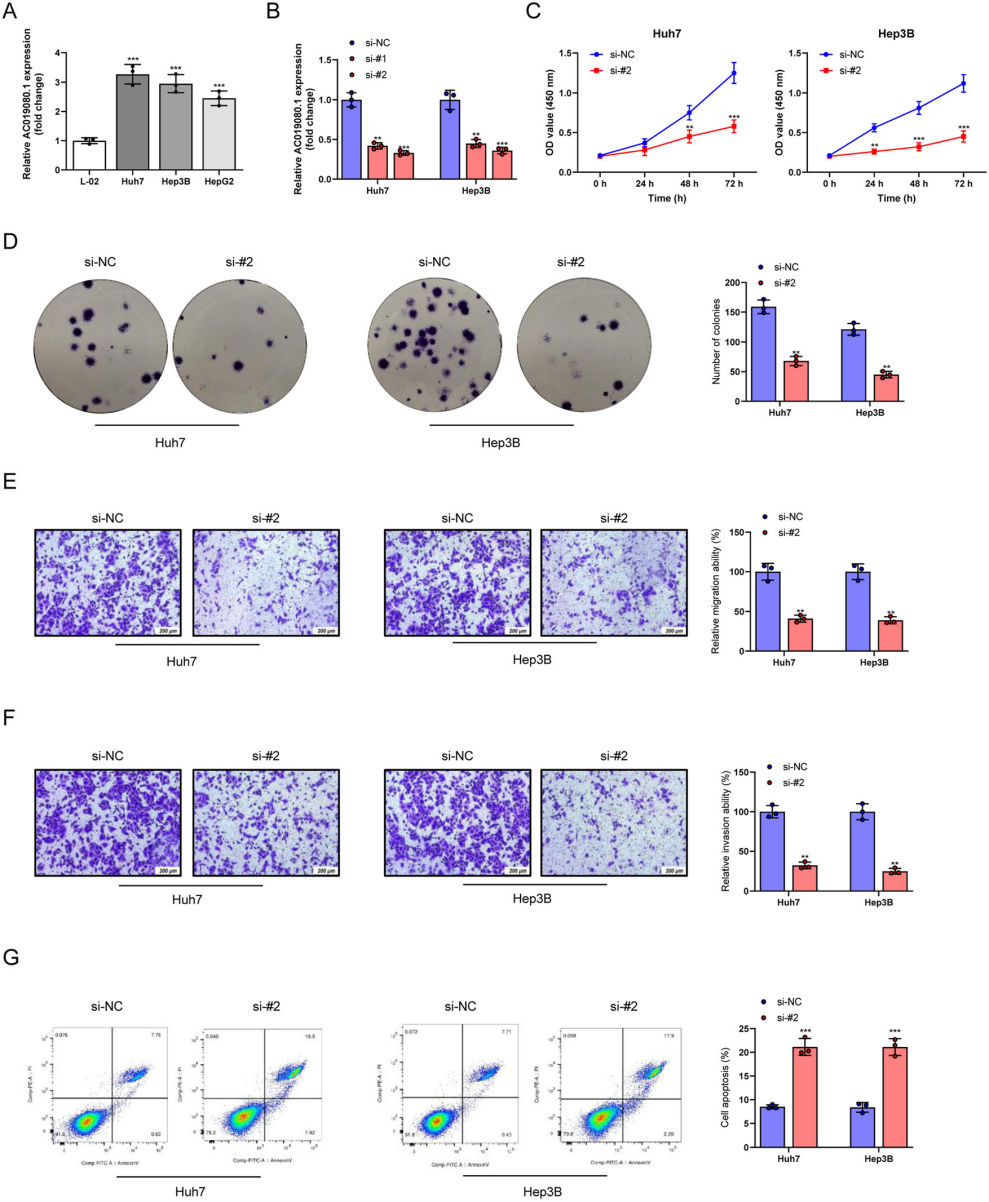
Knockdown of AC019080.1 inhibits HCC cell progression. (A) qRT-PCR was utilized to detect the expression of AC019080.1 in normal cell line L-02 and HCC cell lines (Huh7, Hep3B, and HepG2). (B) The transfection efficiency of AC019080.1 was evaluated by qRT-PCR. (C) CCK-8 assay was carried out to assess the effect of AC019080.1 knockdown on the proliferation potential of HCC cells. (D) The clonogenic capacity of the indicated groups was assessed by colony formation assay. (E, F) Transwell assays were employed to detect the impact of AC019080.1 knockdown on the migration and invasion abilities of HCC cells. (G) Flow cytometry was used to examine the effect of AC019080.1 knockdown on the apoptosis of HCC cells. Data are presented as mean ± standard deviation (SD) from three independent experiments. ^**^ *P* < 0.01, ^***^ *P* < 0.001 versus si-NC group. Note: HCC, hepatocellular carcinoma; qRT-PCR, quantitative real-time polymerase chain reaction.

Next, the role of lncRNA AC019080.1 in HCC was explored through multiple experimental approaches. First, qRT-PCR analysis revealed that AC019080.1 was significantly upregulated in HCC cell lines (Huh7, Hep3B, and HepG2) compared to the normal hepatic cell line L-02 (*P* < 0.001) (Figure 10A). To investigate its functional role, specific siRNAs (si-#1 and si-#2) targeting AC019080.1 were designed. qRT-PCR results confirmed successful knockdown, with si-#2 showing higher efficiency, and it was selected for subsequent experiments (*P* < 0.01, *P* < 0.001) (Figure 10B). Loss-of-function assays were conducted to explore its functional impact. The CCK-8 assay demonstrated that knockdown of AC019080.1 significantly inhibited cell proliferation in Huh7 and Hep3B cells compared to the si-NC group (*P* < 0.01, *P* < 0.001) (Figure 10C). Colony formation assays revealed that AC019080.1 knockdown impaired the clonogenic ability of both Huh7 and Hep3B cells (*P* < 0.001) (Figure 10D). Transwell assays indicated that silencing AC019080.1 significantly reduced the migratory and invasive capabilities of Huh7 and Hep3B cells compared to the si-NC group (*P* < 0.01) (Figure 10E and F). Finally, flow cytometric analysis revealed a significant increase in apoptosis rates in both Huh7 and Hep3B cells following AC019080.1 knockdown (*P* < 0.001) (Figure 10G). In conclusion, lncRNA AC019080.1 plays a critical role in regulating several biological behaviors in HCC cells.

### 3.8 Knockdown of AC019080.1 suppresses HCC cell progression by inhibiting the Wnt/β-catenin signaling pathway

**Figure 11:**
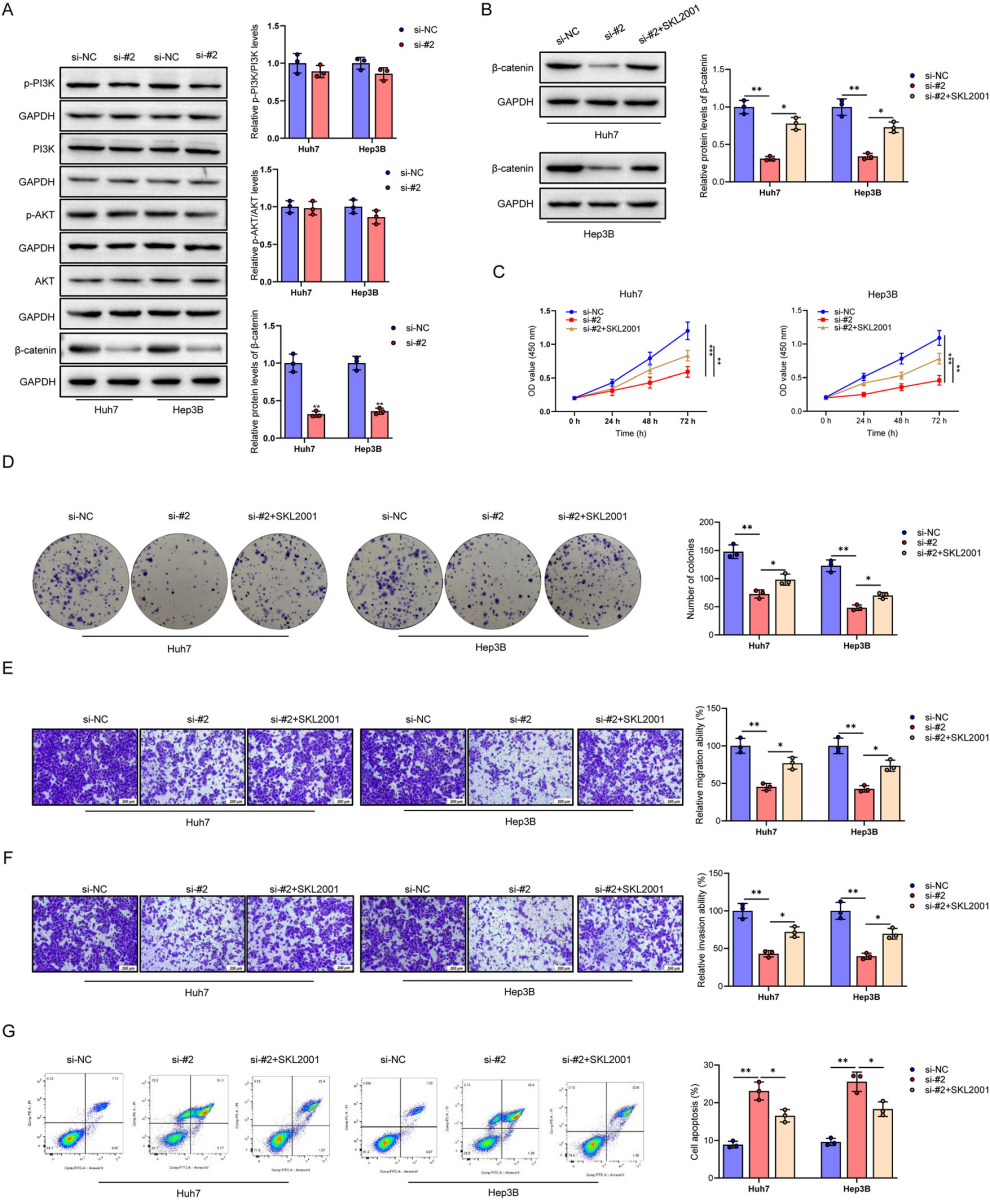
Knockdown of AC019080.1 suppresses HCC cell progression by inhibiting the Wnt/β-catenin signaling pathway. (A) Western blot was used to detect the expression levels of p-PI3K, PI3K, p-AKT, AKT, and *β*-catenin in HCC cell lines (Huh7, Hep3B). (B) Activation of the Wnt/β-catenin pathway was assessed by Western blot detection of *β*-catenin expression in HCC cells treated with the agonist SKL2001. (C) The impact of AC019080.1 knockdown on HCC cell proliferation was assessed by CCK-8 assay, both in the absence and presence of SKL2001. (D) colony formation assays were conducted to evaluate the effects of AC019080.1 knockdown and SKL2001 on the clonogenic potential of HCC cells. (E, F) Migration and invasion of AC019080.1-knockdown HCC cells were assessed by Transwell assays following treatment with SKL2001. (G) Flow cytometry was used to evaluate the effect of AC019080.1 knockdown on apoptosis in HCC cells, with or without activation of the Wnt/β-catenin pathway by the agonist SKL2001. Data are presented as mean ± standard deviation (SD) from three independent experiments. ^**^ *P* < 0.05, ^**^ *P* < 0.01, ^***^ *P* < 0.001 versus si-NC or si-#2 group. Note: HCC, hepatocellular carcinoma; qRT-PCR, quantitative real-time polymerase chain reaction.

Given that the Wnt/β-catenin and PI3K/AKT signaling pathways are well-established key regulators in HCC development and progression [27], we investigated whether AC019080.1 exerts its effects through these pathways. First, we examined the expression levels of key proteins in the Wnt/β-catenin and PI3K/AKT pathways in HCC cells (Huh7 and Hep3B) following AC019080.1 knockdown using Western blot analysis. The results revealed a significant suppression of the Wnt/β-catenin pathway upon AC019080.1 silencing, while the activity of the PI3K/AKT pathway remained largely unchanged (*P* < 0.01) (Figure 11A). To further validate the involvement of Wnt/β-catenin signaling, rescue experiments were conducted using SKL2001, a known activator of the Wnt/β-catenin pathway [28]. Western blot data demonstrated that AC019080.1 knockdown markedly reduced *β*-catenin expression in Huh7 and Hep3B cells, and this reduction was effectively reversed by SKL2001 treatment (*P* < 0.05, *P* < 0.01) (Figure 11B). Subsequently, a series of functional assays were carried out to determine whether the biological effects of AC019080.1 are mediated via Wnt/β-catenin signaling. CCK-8 and colony formation assays, which measure proliferative capacity, showed that silencing AC019080.1 significantly impaired HCC cell proliferation, and this inhibition was partially reversed by SKL2001 treatment (*P* < 0.05, *P* < 0.01, *P* < 0.001) (Figure 11C and D). Transwell assays indicated that the suppression of migratory and invasive abilities caused by AC019080.1 knockdown was partially rescued by SKL2001 treatment (*P* < 0.05, *P* < 0.01) (Figure 11E and F). Furthermore, flow cytometric analysis showed a significant increase in apoptosis upon AC019080.1 silencing, the effect that was attenuated in the presence of SKL2001 (*P* < 0.05, *P* < 0.01) (Figure 11G). Collectively, these findings indicate that knockdown of AC019080.1 suppresses HCC progression by inhibiting the Wnt/β-catenin signaling pathway.

## 4 Discussion

HCC remains a significant challenge in oncology, marked by high heterogeneity and poor prognosis. Our research explored the role of CRR-lncRNAs in HCC, revealing notable associations and novel insights. From the TCGA database, 24 CRR-mRNAs and 433 CRR-lncRNAs linked to HCC were identified. Using one-way COX regression, 46 PCRR-lncRNAs were screened, all of which were upregulated in HCC compared to normal tissues. Among these, AC019080.1 (HR = 4.31) and MCM3AP-AS1 (HR = 2.88) emerged as core PCRR-lncRNAs, showing significant positive co-expression with other PCRR-lncRNAs and higher expression in HCC tissues and cell lines. Functional assays demonstrated that knockdown of lncRNA AC019080.1 inhibited HCC cell proliferation, migration, and invasion, and induced apoptosis by inhibiting the Wnt/β-catenin signaling pathway.

Prior studies have highlighted the impact of circadian rhythm disruption in HCC. Zhu et al. [29] reported that abnormal circadian gene expression is closely associated with tumor malignancy and poor prognosis. Our findings of aberrantly expressed CRR-mRNAs and CRR-lncRNAs in HCC align with these observations, suggesting that dysregulation of the CRRG network is a key factor in HCC development. In a study by Padilla et al. [30], chronic circadian rhythm disruption in a humanized mouse liver model could induce NASH-related carcinogenesis, and the gene expression characteristics of the HCC transcriptome in mice with circadian rhythm disorder closely resembled those of patients with the worst prognosis, highlighting the significant influence of circadian rhythm on HCC progression. Mteyrek et al. [31] demonstrated that suppression of Per and Cry genes disrupted cancer cell circadian rhythms, promoting carcinogenesis. Zhu et al. [32] highlighted the role of genetic abnormalities, such as copy number variations, in prognosis, further complementing research on CRR-lncRNAs. Our identification of a substantial number of CRR-lncRNAs expands on this concept, suggesting that lncRNAs may also contribute to this process. For instance, Cui et al. [33] reported that the lncRNA HULC could upregulate the circadian oscillator CLOCK in hepatoma cells, perturbing the circadian rhythm and promoting hepatocarcinogenesis, further supporting the involvement of lncRNAs in the circadian-related development of HCC.

Regarding the TME, Xiao et al. [34] emphasized that immune cells within the TME interact with tumor cells, influencing tumor progression and treatment response. In the present study, three distinct HCC molecular subtypes with unique immune cell infiltration patterns and survival prognoses were identified. Bao et al. [35]demonstrated the relationship between lncRNAs and the immune landscape in other cancers, which supports our findings in HCC. The Cluster2 subtype exhibited a better prognosis and higher immune-related scores, consistent with the understanding that a more active immune microenvironment contributes to improved patient outcomes [36]. Research in head and neck squamous cell carcinoma (HNSCC) has shown that CCRG risk models are associated with the immune landscape. Specifically, the high-risk group in HNSCC showed increased abundance of activated mast cells, dendritic cells, and neutrophils, which were positively correlated with poor overall survival. This suggests commonalities in the relationship between circadian rhythms, the immune microenvironment, and prognosis across different cancers, providing valuable insights into the TME’s role in HCC.

The role of lncRNAs in cancer has been extensively researched. Shetty et al. [37] and Alshahrani et al. [38] reported that lncRNAs are involved in key tumor-related processes such as metabolic reprogramming and epithelial-mesenchymal transition. Our finding that AC019080.1 promotes HCC cell proliferation, migration, and invasion aligns with these reports, suggesting that this lncRNA may function as an oncogene. Wang et al. [39] described the role of angiogenesis-related lncRNAs in HCC, which is similar to the function of AC019080.1. Previous studies have shown that lncRNAs regulate tumor cell metabolism by modulating metabolic enzymes [40], [41], [42], [43], [44], [45]. It is plausible that AC019080.1 may also influence HCC cell metabolism, although this requires further investigation. For example, a study on melatonin in HCC found that melatonin inhibited HCC progression by regulating the lncRNA-CPS1-IT-mediated HIF-1α inactivation pathway, affecting epithelial-mesenchymal transition and HCC metastasis. This further supports the diverse regulatory roles of lncRNAs in HCC. Notably, our mechanistic investigations revealed that knockdown of AC019080.1 suppressed HCC progression through inhibition of the Wnt/β-catenin signaling pathway, as evidenced by reduced levels of *β*-catenin upon AC019080.1 knockdown and the reversal of this effect by the Wnt/β-catenin pathway agonist SKL2001. Given the well-established role of Wnt/β-catenin signaling in driving cell proliferation, stemness, and metastasis in HCC [46], the inhibition of the Wnt/β-catenin pathway observed upon AC019080.1 knockdown provides a possible mechanism for the oncogenic function of AC019080.1 itself. Importantly, while AC019080.1 significantly modulated Wnt/β-catenin activity, our data indicated that it had no significant influence on the PI3K/AKT signaling pathway – another key pathway frequently dysregulated in HCC [47]. This selective regulation highlights the functional specificity of AC019080.1 in HCC pathogenesis and suggests potential for targeted therapies. Specifically, targeting AC019080.1 may allow for more precise treatment by affecting a specific cancer-related pathway, without broadly interfering with other important cellular signals.

Nevertheless, this study has several limitations. Relying solely on the TCGA database for samples and data may introduce selection bias, and external validation using independent cohorts or datasets is essential. Furthermore, although our *in vitro* functional assays have demonstrated that AC019080.1 promotes HCC progression, at least in part, through modulation of the Wnt/β-catenin signaling pathway, the absence of ex vivo evidence means that the broader molecular mechanisms – particularly its downstream targets, interacting partners, and regulatory networks – remain to be fully elucidated. Future studies should extend these findings by identifying the interactions of AC019080.1 with other molecules in HCC cells and validating the functional roles and mechanisms of additional candidate lncRNAs identified in this study. Additionally, examining the role of these lncRNAs in the TME through *in vivo* models could provide valuable insights for developing targeted therapies. A study by the Liu Chang team from China Pharmaceutical University demonstrated that POLB could drive HCC progression in a circadian rhythm-dependent manner by mediating the demethylation of Per1’s 5′ UTR [48]. This finding serves as an example of an in-depth exploration of the relationship between genes, circadian rhythms, and HCC progression, indicating the potential of exploring the role of lncRNAs in similar contexts to provide new avenues for HCC treatment.

In summary, this study identified novel CRR-lncRNAs associated with HCC and highlights the critical role of lncRNA AC019080.1 in HCC cell progression. These findings not only expand our understanding of the molecular mechanisms underlying HCC but also provide potential targets for the development of innovative diagnostic and therapeutic strategies. Despite the existing limitations, this study lays a foundation for future research in this field, paving the way for more in-depth investigations into the complex relationship between circadian rhythms, lncRNAs, and HCC.

## 5 Conclusions

In conclusion, leveraging the TCGA database, this study identified correlations between HCC, 24 CRR-mRNAs, and 433 CRR-lncRNAs. It revealed that lncRNA AC019080.1 plays a pivotal role in HCC cell progression, offering potential new diagnostic and therapeutic strategies.

## Supplementary Material

Supplementary Material

Supplementary Material

Supplementary Material

Supplementary Material

Supplementary Material

Supplementary Material

Supplementary Material
