# Supplementary material for: Circadian rhythm-based prognostic features predict immune infiltration and tumor microenvironment in molecular subtypes of hepatocellular carcinoma
Source: Open Life Sci. 2026 Jan 1;20(1):20251208. doi: 10.1515/biol-2025-1208 (PMC13011610; doi:10.1515/biol-2025-1208)
Supplement: Supplementary file 1 — Supplementary Material [file j_biol-2025-1208_suppl_001.docx]

**Captions for the Supplementary Tables:**

Table S1. RNA transcriptome expression matrix of hepatocellular carcinoma from TCGA database.

Table S2. lncRNA transcriptome expression matrix of hepatocellular carcinoma from TCGA database.

Table S3. mRNA transcriptome expression matrix of hepatocellular carcinoma from TCGA database.

Table S4. A matrix of 24 CRR-mRNAs based on the mRNA’s matrix of HCC and literature reports.

Table S5. Expression matrix of 433 CRR lncRNAs.

Table S6. The co-expression relationship between CRR-mRNAs and CRR-lncRNAs.
